# Higher BMI increases risk of stoma-site incisional hernia and other complications following diverting loop ileostomy and reversal: a systematic review and meta-analysis

**DOI:** 10.1007/s00464-025-11887-y

**Published:** 2025-06-26

**Authors:** Kaiser O’Sahil Sadiq, Swetha Lakshminarayanan, Patricia Ruiz Cota, Eugenia Marquez Castillo

**Affiliations:** 1 Department of Surgery, UC San Diego, San Diego, CA USA; 2 Department of Surgery, Abington Jefferson Hospital, Philadelphia, PA USA

**Keywords:** Morbidity, Complications, Obesity, BMI, Diverting loop ileostomy, Stoma

## Abstract

**Background:**

Diverting loop ileostomies (DLI) are commonly used to protect high-risk anastomoses. While a higher body mass index (BMI) is known to increase postoperative morbidity, its specific effect on DLI-related complications is less well understood.

**Methods:**

This systematic review and meta-analysis followed PRISMA guidelines, and Cochrane Library, Embase, PubMed, and Google Scholar were searched for English publications without time restrictions. Statistical analysis was performed using Meta-Mar v3.5.1.

**Results:**

Out of 586 studies yielded and 6 manually retrieved, 26 were included, totaling 5,141 patients (53.5% male). The mean ages were 35.0–65.3 years, and the mean BMI was 19.8–27.1 kg/m^2^. Higher BMI increased stoma-site incisional hernia in 100% (*n* = 7) with a standardized mean difference of 0.88 (95% CI, 0.34–1.42). Two studies (40%) reported increased peristomal skin complications. One study each (100%) found increased risk for delayed reversal, stoma outlet obstruction, stoma-specific morbidity score, and longer operative time. Elevated BMI increased anastomotic leakage in 2 studies (50%), surgical site infection in 1 (33%), permanent stoma in 1 (20%), and overall complications in 2 (67%). However, no significant BMI differences were seen for high-output ileostomies, stoma retraction, parastomal hernia, complications after reversal, length of hospital stay, or mortality. BMI > 30 was associated with higher complication risks overall (OR [95% CI], 2.01 [1.11–3.64]), but BMI > 25 showed no significant difference except for stoma-site incisional hernia (OR [95% CI], 4.66 [3.54–6.14]) and parastomal hernia (OR [95% CI], 2.41 [1.70–3.40]).

**Conclusion:**

Increased BMI is a risk factor for certain DLI-related complications, particularly stoma-site incisional hernia. If feasible, mesh placement at the ileostomy site should be strongly considered for patients with a BMI > 25 to reduce the risk of stoma-site incisional hernia. Weight loss prior to DLI and reversal should be encouraged.

Fecal diversion is routinely employed in cases of high-risk anastomoses to mitigate the consequences of anastomotic leakage [1,2]. Among the various techniques available, diverting loop ileostomies (DLI) are most frequently utilized due to their relative ease of construction and reversal [2, 3], as well as their association with fewer complications and improved quality of life [4]. The utilization of DLI varies significantly depending on the surgical context, with reported rates of 2–39% in inflammatory bowel disease (IBD) resections and up to 91% in anterior resections [5, 6]. However, complications associated with DLI are not uncommon, occurring in up to 64% of cases [7], with a reported mortality rate of 0.4% [8]. Obesity is a well-established risk factor for a range of postoperative complications [9, 10], including surgical site infections [11, 12], wound dehiscence [12], and incisional hernia formation [13]. Furthermore, obesity has been demonstrated to increase the risk of anastomotic leakage [14–16], potentially leading to a greater reliance on fecal diversion strategies in this patient population. Despite this, there remains a paucity of data specifically examining the impact of elevated body mass index (BMI) on complications related to DLI.

To address this gap in the literature, a systematic review and meta-analysis were conducted to evaluate the influence of BMI on postoperative complications following DLI and its reversal. This review aims to provide evidence-based insights to inform shared decision-making regarding the risks and benefits of this commonly performed procedure in patients with elevated BMI.

## Methods

### Study design

This systematic review and meta-analysis were conducted following the PRISMA guidelines [17]. The study protocol was registered on PROSPERO under the ID CRD42024581114 [18]. Two independent reviewers screened and appraised the quality of the selected studies, followed by data extraction using a standardized table. Discrepancies between reviewers were resolved through discussion and if necessary, a third reviewer was consulted to reach a consensus.

### Search strategy

A comprehensive search of studies published up to August 12, 2024 was performed across the Cochrane Library, EMBASE, PubMed, PubMed Central, and Medline databases. The search string utilized was as follows: (“loop ileostomy” OR “loop ileostomies” OR “diverting ileostomy” OR “diverting ileostomies” OR “temporary ileostomy” OR “temporary ileostomies”) AND (obesity OR obese OR BMI OR body mass index). Additional manual searches were conducted using Google Scholar and by reviewing reference lists of relevant articles.

### Inclusion and exclusion criteria

Eligible studies included clinical trials, case series, case–control studies, and cohort studies published in English. Studies with other designs, such as animal studies, in vitro studies, case reports, conference abstracts, irretrievable studies, or those published in languages other than English, were excluded. Studies lacking BMI-related comparisons for complications or those without data limited to diverting loop ileostomies were also excluded. Only human studies in English were considered, with no restrictions based on publication date.

### Quality assessment

The quality of cohort and case–control studies was evaluated using the Newcastle–Ottawa Scale (NOS) [19], with thresholds defined by the Agency for Healthcare Research and Quality (AHRQ) [20]. Randomized controlled trials (RCTs) were assessed using the revised Cochrane Risk of Bias Tool (RoB 2) [21]. While all studies were included in the review, only RCTs with a low risk of bias and other studies deemed to be of good quality were included in the meta-analysis.

### Extracted data and variables

Data extracted from the studies included sample size, mean age, gender distribution, BMI, follow-up duration, intraoperative details such as concomitant procedures, surgical approach, urgency, operative time, length of stay (LOS), time to reversal, method of skin closure, and the distribution of patients with complications and elevated BMIs.

### Meta-analysis

For studies reporting median values and ranges due to skewed data distributions, the Method for Unknown Non-Normal Distributions approach was employed to estimate mean and standard deviation [22]. Statistical analysis and visualization were performed using Meta-Mar v3.5.1 [23]. Standardized mean differences (SMD) were calculated from studies that reported the mean BMI at which complications did and did not occur. Pooled odds ratios (OR) were calculated for studies that reported the number of patients in the various BMI cohorts that did or did not develop complications. SMD and OR were calculated using a Hartung–Knapp-adjusted random-effects model with the inverse variance method, and results were visualized using forest plots and box plots. Effect sizes were estimated using Hedge’s g, while heterogeneity was assessed using tau and *I*^2^. The restricted maximum-likelihood estimator was used for tau^2^, and the Q-Profile method was applied to calculate confidence intervals for tau^2^ and tau.

## Results

### Search results and demographics

**Fig. 1:**
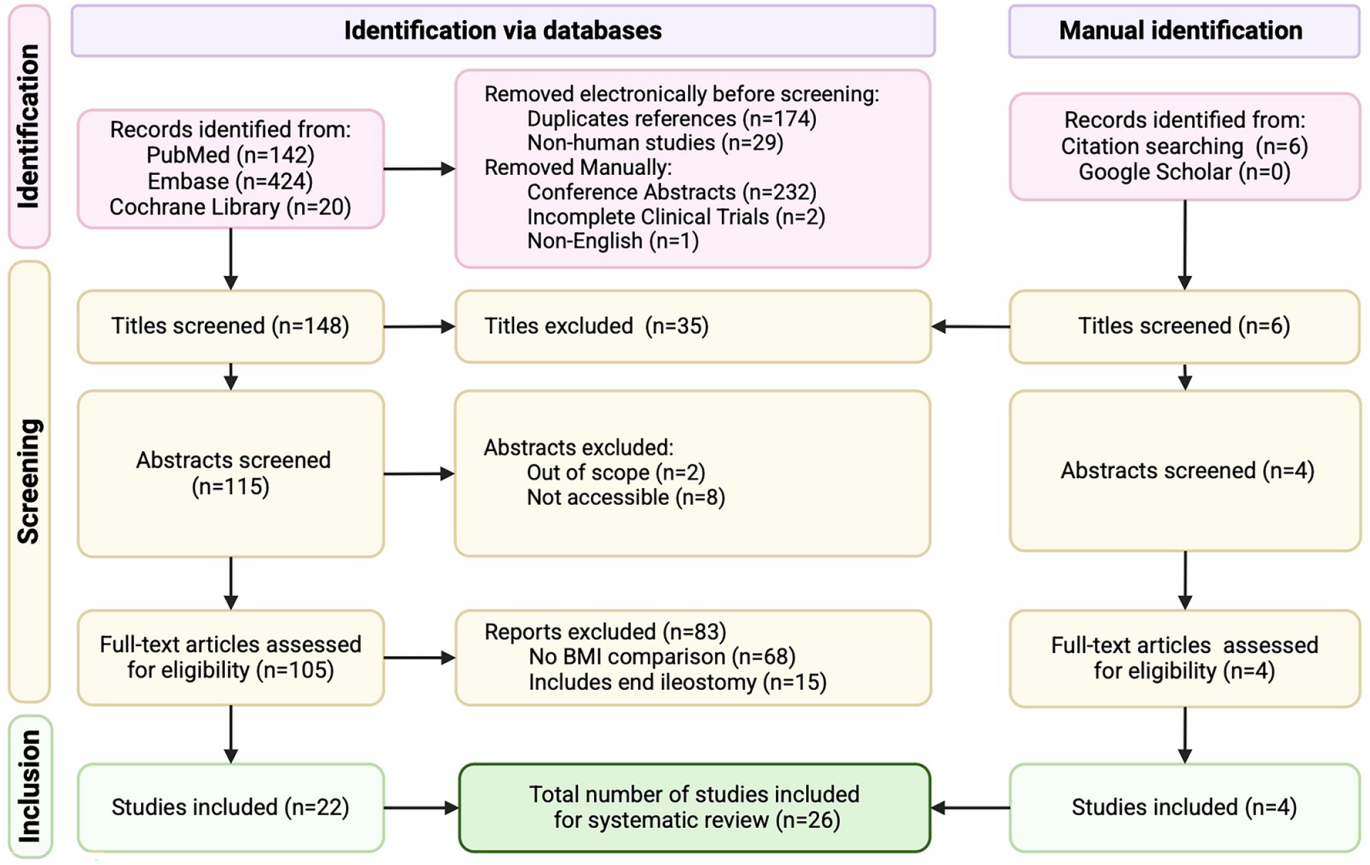
PRISMA (Preferred Reporting Items for Systematic Reviews and Meta-Analyses) flowchart of study selection

**Fig. 2:**
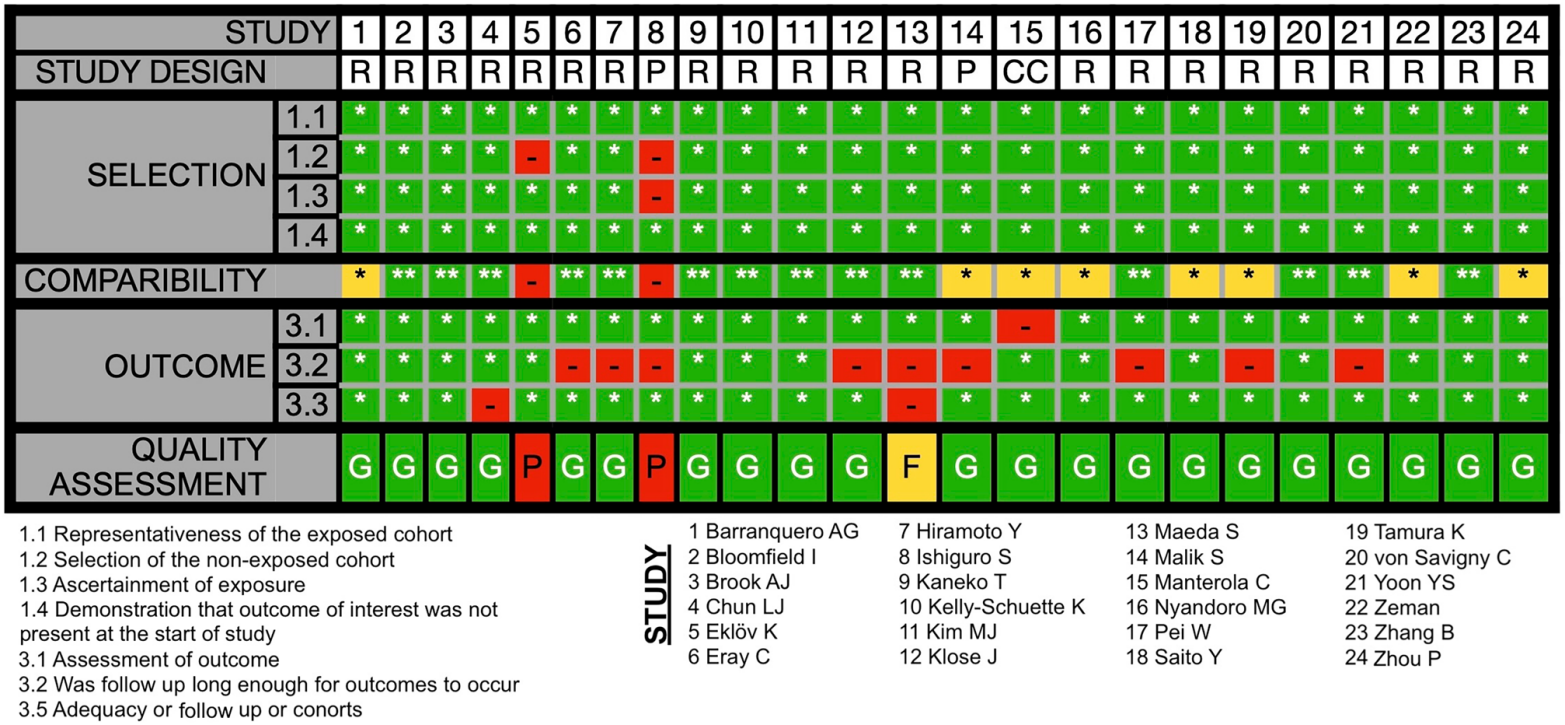
Chart depicting the quality of included cohort and case–control studies critically appraised using the Newcastle–Ottawa Scale and Agency for Healthcare Research and Quality thresholds. (Study Designs) *CC* Case–Control, *P* Prospective cohort *R* Retrospective cohort (Quality assessments) *F* Fair, *G* Good, *P* Poor

**Fig. 3:**
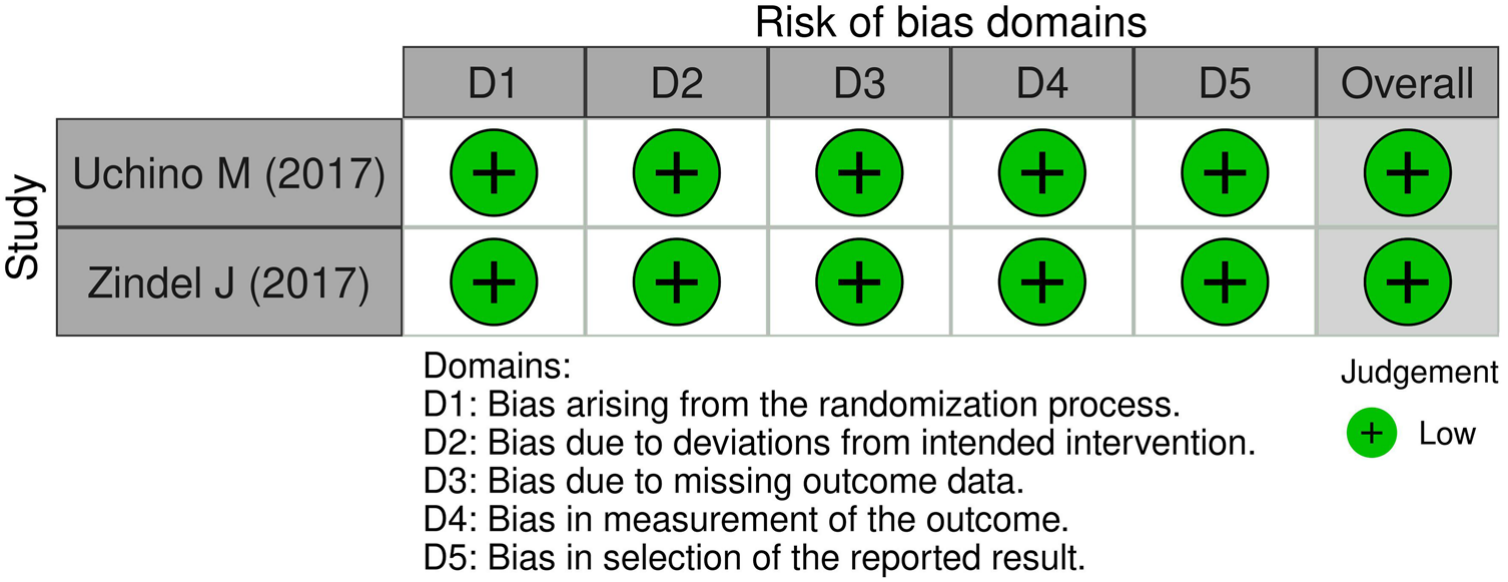
Traffic light plot depicting the quality of included RCTs critically appraised using the RoB2 tool. *RCT* Randomized controlled trial, *RoB2* A revised tool for assessing risk of bias in randomized trials

The initial search strategy identified 586 articles, with an additional six references identified through manual searches of Google Scholar and reference lists. After exclusion of 566 articles for various reasons, as illustrated in Fig. 1, 26 articles underwent quality assessment [4, 5, 7, 16, 24–45]. These included 21 retrospective cohorts [5, 7, 16, 24–29, 31–34, 37–43, 45], two prospective cohorts [30, 35], and one case–control study [36], with methodological quality rated as good in 87.5% (*n *= 21) [5, 7, 16, 24–26, 28, 29, 31–33, 35–43, 45], fair in 4.2% (*n* = 1) [34], and poor in 8.3% (*n* = 2) [27, 30], shown in Fig. 2. Both randomized controlled trials (RCTs) were found to have a low risk of bias (Fig. 3) [4, 44].

As summarized in Table 1, the sample sizes of the included studies ranged from 4 to 972 patients (median, 171), encompassing a total of 5,141 patients (53.5% male). The mean patient age ranged from 35.0 to 65.3 years, and the mean BMI ranged from 19.8 to 27.1 kg/m^2^. The follow-up duration averaged between 5.5 and 49.5 months. Most studies did not directly test the association with BMI, but the reported data were used to answer this secondarily.

**Table 1:** Study design and demographic data for included studies

First Author	Study Design	Sample Size	Avg Age (y)	% M	% F	Avg BMI (kg/m^2^)	Follow-Up (mo)
Barranquero AG	RC	129	67* (55.5–75)^IQR^	67.4	32.6	26.3* (24–28.5)^IQR^	37* (22–57)^IQR^
Bloomfield I	RC	171	63 ± 14.7	52.6	47.4	26.3 ± 5.87	–
Brook AJ	RC	193	66* (20–92)^r^	59.6	40.4	25* (16–44)^r^	20.5* (0–69)^r^
Chun LJ	RC	123	49, 51* (12–79)^r^	56.1	43.9	–	22, 17* (1–65)^r^
Eklöv K	RC	216	62, 63* (55–70)^IQR^	60	40	26, 25*, (23–28)^IQR^	30*
Eray IC	RC	66	58.5 (23–83)^r^	75.8	24.2	26.8	–
Hiramoto Y	RC	45	59.6	46.7	53.3	21.1 (15.6–29.1)^r^	–
Ishiguro S	PC	4	49.8	75	25	28.5	–
Kaneko T	RC	134	65* (27–85)^r^	62.7	37.3	22.1* (16.1–36.7)^r^	47* (8–30)^r^
Kelly-Schuette K	RC	243	56.5 ± 17.2	50.6	49.4	27.5 ± 6.8	49.5* (1–80)^r^
Kim MJ	RC	673	58.6	–	–	24	–
Klose J	RC	972	61.9 ± 11.4	67.9	32.1	–	–
Maeda S	RC	185	62* (27–83)^r^	70.8	29.2	21.7* (15.3–43.3)^r^	–
Malik S	PC	100	35 ± 5.8	76	24	26.3 ± 4.3	42
Manterola C	CC	39	71.3 ± 7.1	–	–	27.3 ± 19.8	–
Nyandoro MG	RC	106	59 ± 14.8	67.9	32.1	27* (6.4)^IQR^	–
Pei W	RC	242	58.3	66.5	33.2	24.2*	–
Saito Y	RC	82	64* (32–86)^r^	68.3	31.7	23.4* (16.5–37.6)^r^	–
Tamura K	RC	230	65* (35–83)^r^	71.3	28.7	22.2* (15.9–32.4)^r^	–
Uchino M	RCT	257	42	64.2	35.8	19.8	–
von Savigny C	RC	300	60.4 ± 14.4 (21–90)^r^	61.7	38.3	24.2 ± 4.7 (13–43)^r^	48.8 ± 41.1 (0–148)^r^
Yoon YS	RC	91	30* (17–79)^r^	52.7	47.3	21.8* (14.9–40.9)^r^	–
Zeman M	RC	101	62.0 ± 10.6, 66* (55.5–75)^IQR^ (20–92)^r^	–	–	26.9 ± 4.4 26* (23.8–29.7)^IQR^ (18.4–42.6)^r^	–
Zhang B	RC	185	60* (25–81)^r^	60.5	39.5	24.8* (18.63–36.33)^r^	40* (6–07)^r^
Zhou P	RC	176	50.3, 53*	53	47	27.1, 26* (14.7–46)^r^	16.9 (0.3 -46.9)^r^
Zindel J	RCT	78	62.1	71.9	28.1	26.1, 26*	5.5

All unmarked values denote mean ± standard deviation, *median, ^r^range, ^IQR^interquartile range

*CC* Case–control, *F* Female, *M* Male, *PC* Prospective cohort, *RC* Retrospective cohort, *RCT* Randomized control trial

### Admission and operative variables

At the time of DLI, concomitant procedures included low anterior resection [4, 7, 27, 28, 38, 40, 42], proctocolectomy [7, 29, 34, 44], partial colectomy [29], sigmoidectomy [32], ileocectomy [5], and Hartmann’s reversal [4]. When reported, the proportion of ileostomies performed electively ranged from 64.4% to 100% [4, 5, 7, 24, 26, 28–30, 39, 41, 44], laparoscopically from 4 to 100% [4, 5, 7, 16, 24, 26–30, 34, 37–40, 43], and via an open approach from 0% to 94.9% [4, 5, 7, 16, 24, 26–30, 34, 37–40, 43]. The mean operative time for the index surgery ranged from 146 to 316.5 min [4, 5, 16, 29, 30, 38, 43, 44], and the average LOS varied between 3 and 26.8 days [4, 5, 26, 29, 35]. The time to DLI reversal ranged from 100 to 300 days [7, 24, 26, 27, 30, 32–34, 36, 39, 41, 43, 45], with a mean operative time for reversal ranging from 62.5 to 87.4 min [32, 36, 37, 41] and an average LOS of 3 to 8.8 days [7, 27, 32, 35–37, 41, 45].

The technique of ileostomy site skin closure was reported in seven studies [24–26, 31, 37, 41, 45], with conventional linear suture used in all seven studies (17–100% of cases) and purse-string closure in five studies (3–68.9% of cases) [24, 31, 37, 41, 45]. A single study each reported triangular-type closure in 78% of cases [31] and closure by secondary intention in 13.1% of cases [45]. Fascial closure was not described, and mesh was not used in any of the included studies. These variables are further detailed in Table 2.

**Table 2:** Operative and admission details reported by included studies

First Author	Concomitant Procedures	Elective/ Urgent/ Emergent	Approach (%)				DLI creation		Avg Time Till Reversal (d)	DLI Reversal		Skin Closure				
			Lap	Conv	Open	NR	Avg Operative Time (min)	Avg LOS (d)		Avg Operative Time (min)	Avg LOS (d)	Conventional Linear	Purse-String	Open	Triangular	NR
Barranquero AG	-	Elective (93.8%) Urgent (6.2%)	32	0	68.2	0	–	–	252* (168–308)^IQR^	–	–	56.6	28.7	0	0	15
Bloomfield I	–	NR	–	–	–	100	–	–	–	–	–	100	0	0	0	0
Brook AJ	–	Elective (88%) Emergent (12%)	27	0	73.5	0	–	3 (1–8)^r^	180 ± 11	–	–	82.9	0	0	0	17
Chun LJ	LAR (57.7%) Proctocolectomy, IPAA (29.3%) Other (13%)	Elective (100%)	26	0	74	0	–	–	214, 140* (56–1258)^r^	–	5.7, 3* (1–45)^r^	–	-	-	-	100
Eklöv K	LAR (100%)	NR	4.6	1.4	94	0	–	–	228, 180* (120–270)^IQR^	–	5.1, 4.0* (3.0–5.0)^IQR^	–	–	–	–	100
Eray IC	LAR (100%)	Elective (100%)	59	12.1	28.8	0	–	–	–	–	–	–	–	–	–	100
Hiramoto Y	Proctocolectomy (17.8%) LAR (46.7%) Partial Colectomy (35.6%)	Elective (64.4%) Emergent (35.6%)	64	0	35.6	0	214.3 (105–374)^r^	26.8 (12–90)^r^	–	–	–	–	–	–	–	100
Ishiguro S	–	Elective (100%)	100	0	0	0	146	–	113.8	–	–	–	–	–	–	100
Kaneko T	–	NR	–	–	–	100	–	–	–	–	–	17	3	0	78	2
Kelly–Schuette K	Sigmoidectomy (19%)	NR	–	–	–	100	–	–	100* (33–1119)^r^	87.4 ± 39.9	3* (1–25)^r^	–	–	–	–	100
Kim MJ	–	NR	–	–	–	100	–	–	183 (30–690)^r^	–	–	–	–	–	–	100
Klose J	–	NR	4	1.1	94.9	0	240.7 ± 79.5	–	–	–	–	–	–	–	–	100
Maeda S	Proctocolectomy (100%) with—Coloanal anastomosis (27.6%)—Colorectal anastomosis (72.4%)	NR	43	0	57.3	0	–	–	124* (43–798)^r^	–	–	–	–	–	–	100
Malik S	–	NR	–	–	–	100	–	8 ± 3.5	–	–	8 ± 3.53	–	–	–	–	100
Manterola C	–	NR	–	–	–	100	–	–	300 ± 21	62.5 ± 10.6	3.8 ± 0.1	–	–	–	–	100
Nyandoro MG	–	NR	–	–	–	100	–	–	–	64.8 ± 29.7	5.4 ± 3.2	91.5	8.5	0	0	0
Pei W	LAR (100%)	NR	100	0	0	0	224.7	–	–	–	–	–	–	–	–	100
Saito Y	Rectal Resection (100%)	Creation: Elective (86.6%) Emergent (13.4%) Reversal: Elective (100%)	55	0	45.1	0	–	–	106* (40–293)^r^	–	–	–	–	–	–	100
Tamura K	LAR (100%)	NR	100	0	0	0	–	–	–	–	–	–	–	–	–	100
Uchino M	Proctocolectomy, IPAA	Elective (70.7%) Urgent (29.3%)	–	–	-	100	228.7	–	–	–	–	–	–	–	–	100
von Savigny C	-	Elective (100%)	–	–	–	100	–	–	151.7 ± 132.9 (16–946)^r^	69.3 ± 28.7 (23–282)^r^	8.8 ± 9.8 (2–118)^r^	31.1	68.9	0	0	0
Yoon YS	Ileocectomy (100%) with—Strictureplasty (2.2%)—SB resection (8.8%)—Colon resection (23.1%)—Other (11.0%)—No other procedures (54.9%)	Elective (96.7%) Urgent/Emergent (3.3%)	81	18.7	0	0	167.0* (40.0–512.0)^r^	5.0* (0.0–47.0)^r^	–	–	–	–	–	–	–	100
Zeman M	LAR (100%)	NR	–	–	–	100	–	–	–	–	–	–	–	–	–	100
Zhang B	Intersphincteric Resection	NR	99	0	1.1	0	200* (120–400)^r^	–	113* (14–491)^r^	–	–	–	–	–	–	100
Zhou P	–	NR	–	–	–	100	–	–	134 (17–635)^r^	–	4, 3* (1–20)^r^	25.6	61.4	13.1	0	0
Zindel J	LAR (82.1%) Hartmann Reversal (5.1%) Other (7.7%)	Elective (100%)	56	16.7	26.9	0	316.5	16.8	–	–	–	–	–	–	–	100

All unmarked values denote mean ± standard deviation, *median, ^r^range, ^IQR^interquartile range

Abbreviations: *conv* Converted to open, *d* Day, *IPAA* Ileal pouch-anal anastomosis, *min* Minutes, *NR* Not reported, *lap* Laparoscopic, *LAR* Low anterior resection, *LOS* Length of stay, *SBR* Small bowel resection

### Outcomes and complications

**Fig. 4:**
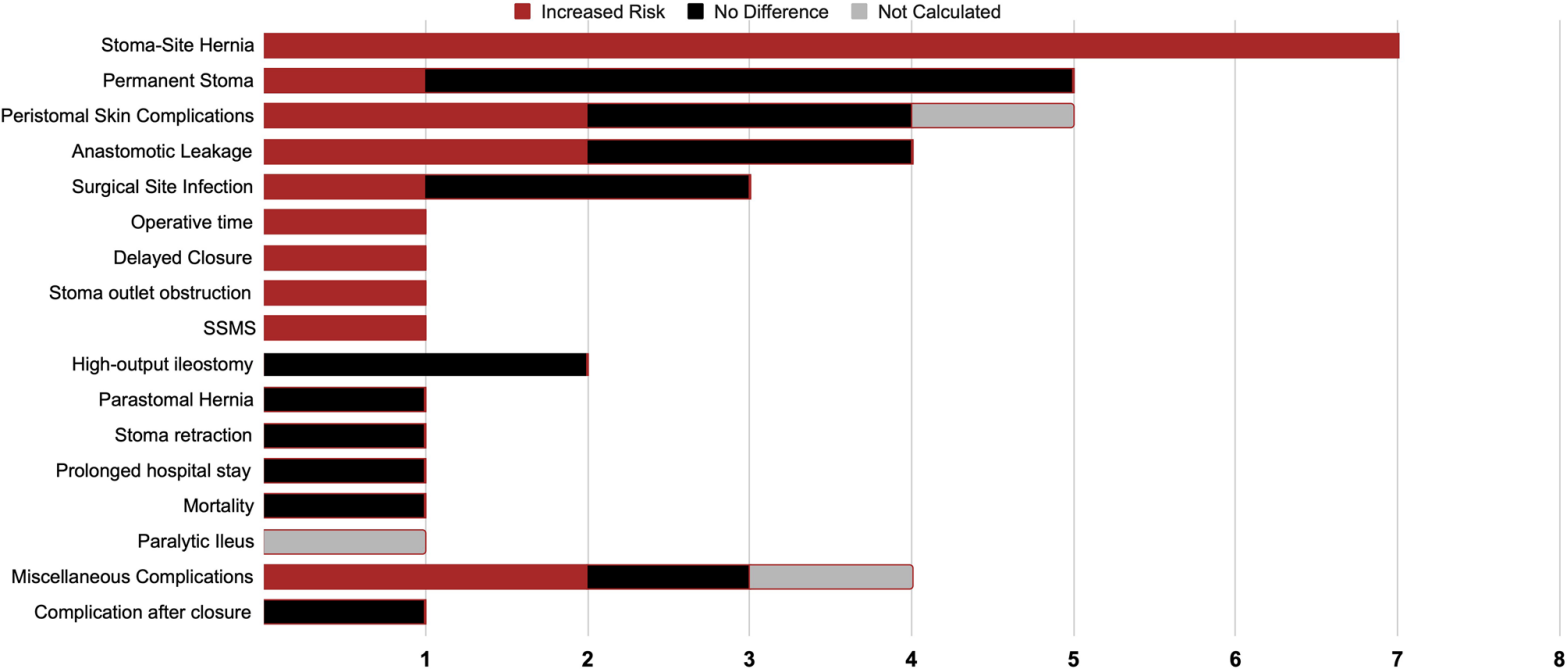
Graph of complications affected by increased BMI. *SSMS* Stoma-specific morbidity scores

Table 3 and Fig. 4 summarize the complications investigated and those found to be influenced by increased BMI. Seven studies (100%) identified elevated BMI as a risk factor for stoma-site incisional hernia [24–27, 31, 32, 45]. One study (20%) found that a BMI > 30 increased the risk of permanent stoma formation [7], while the remaining four studies found no significant difference [28, 33, 42, 43]. Two of five studies (40%) reported increased peristomal skin complications [7, 34], with two specifically finding no difference in the development of peristomal dermatitis [35, 44] and one study not analyzing these outcomes despite available data [30]. An increased risk of anastomotic leakage was observed in two [16, 43] out of four studies (50%) [5, 16, 35, 43]. Only one of these reported the development of anastomotic leakage for ileostomy reversal [35], while the other three evaluated anastomotic leakage following the initial anastomosis [5, 16, 43]. Surgical site infections were increased in one [41] out of three studies (33%) [35, 37, 41].

**Table 3:** Complications investigated by included studies detailing BMI as a risk factor

Outcome	First Author	SD	SS	Mean BMI	BMI risk
Stoma Site Hernia	Kelly–Schuette	RC	243	27.5	Y
	Eklöv	RC	216	26	Y
	Brook	RC	193	25.5	Y
	Zhou	RC	176	27.1	> 30
	Bloomfield	RC	171	26.3	Y
	Kaneko	RC	134	22.5	> 25
	Barranquero	RC	129	26.4	Y
Permanent Stoma	Kim	RC	673	24	N
	Zhang	RC	185	25.1	N
	Chun	RC	123	NR	> 30
	Zeman	RC	101	26.9	N
	Eray	PC	66	26.8	N
Peristomal Skin Complications	Uchino	RCT	257	19.8	N
	Maeda	PC	185	22.2	> 25
	Chun	RC	123	NR	> 30
	Malik	PC	100	26.3	N
	Ishiguro	CS	4	28.5	NA
Anastomotic Leakage	Klose	RC	972	NR	Y
	Zhang	RC	185	25.1	Y
	Malik	PC	100	26.3	N
	Yoon	RC	91	22.5	N
Surgical Site Infection	von Savigny	RC	300	24.2	Y
	Nyandoro	RC	106	NR	N
	Malik	PC	100	26.3	N
Operative time	Pei	RC	242	NR	Y
Delayed Closure	Manterola	CC	39	27.3	> 25
Stoma outlet obstruction	Tamura	RC	230	22.4	> 22.2
SSMS	Zindel	RCT	78	26.1	> 26
High-output ileostomy	Chun	RC	123	NR	N
	Hiramoto	RC	45	21.1	N
Parastomal Hernia	Malik	PC	100	26.3	N
Stoma retraction	Uchino	RCT	257	19.8	N
Prolonged hospital stay	Malik	PC	100	26.3	N
Paralytic Ileus	Ishiguro	CS	4	28.5	NA
Mortality	Malik	PC	100	26.3	N
Overall Complications	Pei	RC	242	NR	NR
	Tamura	RC	230	22.4	N
	Chun	RC	123	NR	> 30
	Saito	RC	82	23.9	> 24.0
Complication after closure	Chun	RC	123	NR	N

All unmarked values denote mean ± standard deviation, *median, ^r^Range, ^IQR^Interquartile range

*CC* Case–control, *N* No statistically significant difference, *Na* Not available, *NR* Not reported, *PC* Prospective cohort, *RC* Retrospective cohort, *RCT* Randomized control trial, *Y* Statistically significant increase

Single studies (100%) reported that higher BMI was associated with prolonged operative times [38], delayed closure [36], increased occurrence of stoma outlet obstruction [40], and higher stoma-specific morbidity scores [4]. No significant increases were observed for mortality [35], prolonged LOS [35], high-output ileostomies [7, 29], stoma retraction [44], or parastomal hernia [35] at higher BMI (100%). Four studies reported grouped complications [7, 38–40], with two (67%) demonstrating an increased risk [7, 39], one finding no difference [40], and the remaining study not analyzing BMI as a variable [38]. One study specifically investigating complications after closure found no significant differences [7]. A single study reported paralytic ileus but did not analyze the data [30].

### Meta-analysis

**Fig. 5:**
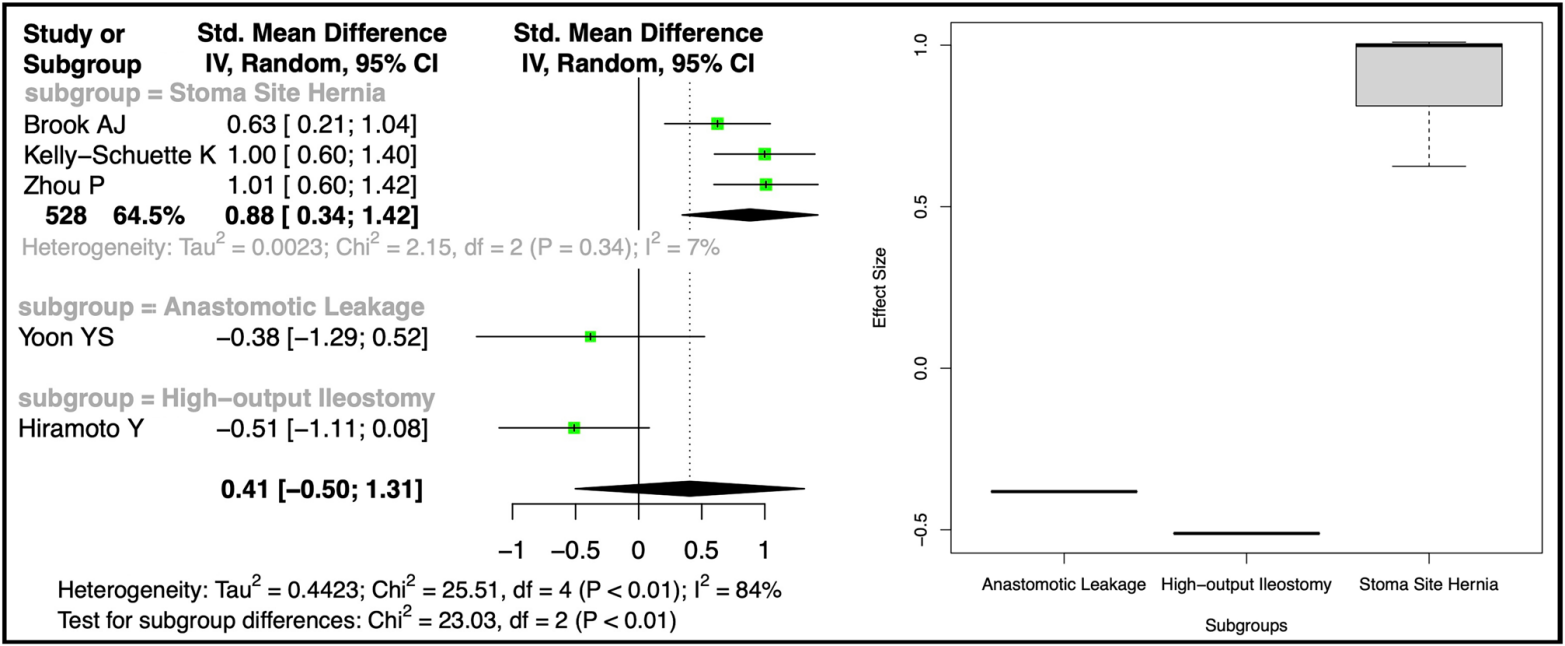
Forest plot depicting the calculated standardized mean difference of BMI between those with and without complications in included studies. The random-effects meta-analysis model (Inverse Variance method) was used. *Cl* Confidence interval, *df* Degrees of freedom, *IV* Inverse variance, *Std* Standard

**Fig. 6:**
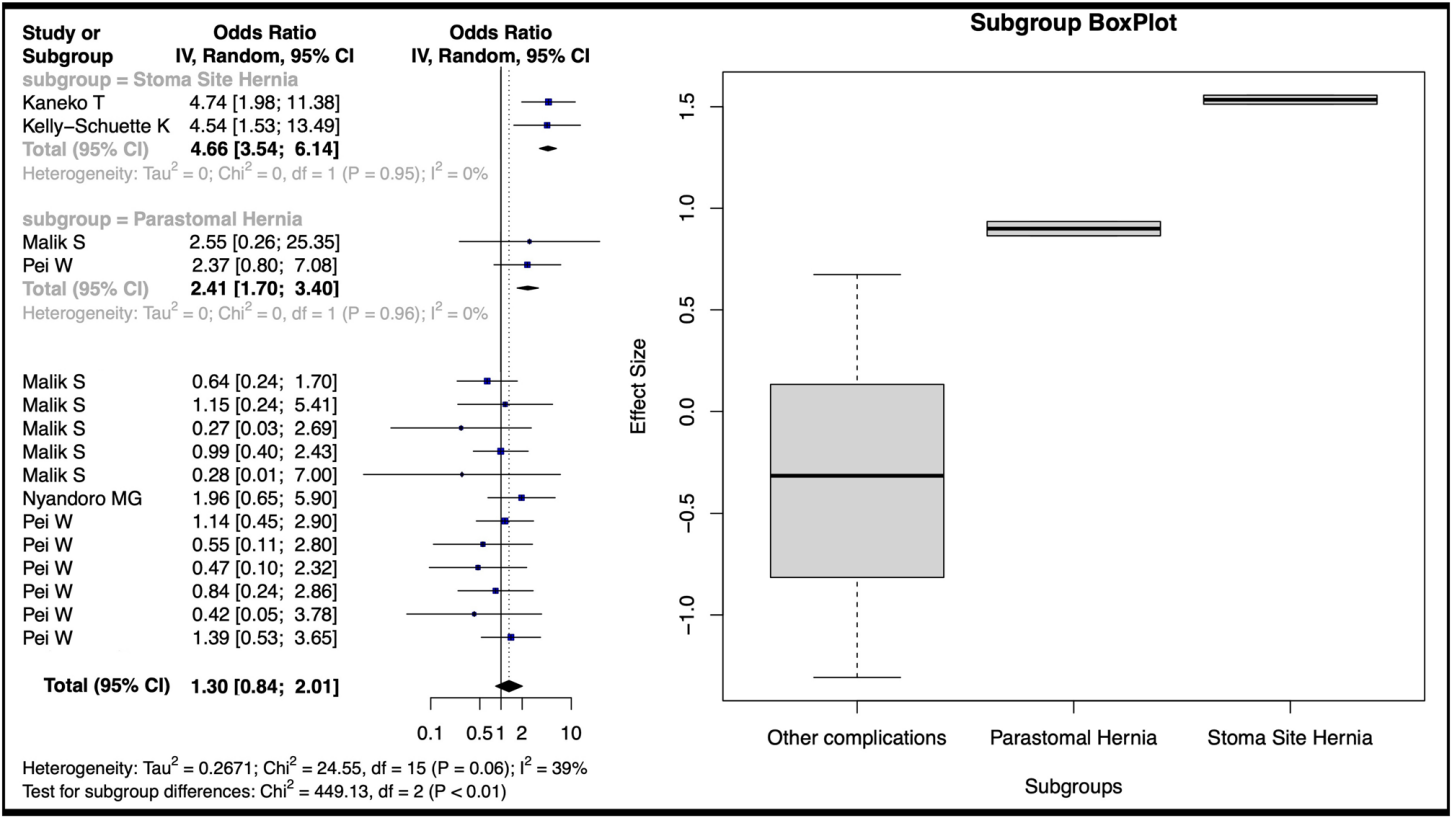
Forest plot of calculated log odds ratios for complications occurring over BMI 25 for included studies. The random-effects meta-analysis model (Inverse Variance method) was used. *Cl* Confidence interval, *df* Degrees of freedom, *IV* Inverse variance, *Std* Standard

**Fig. 7:**
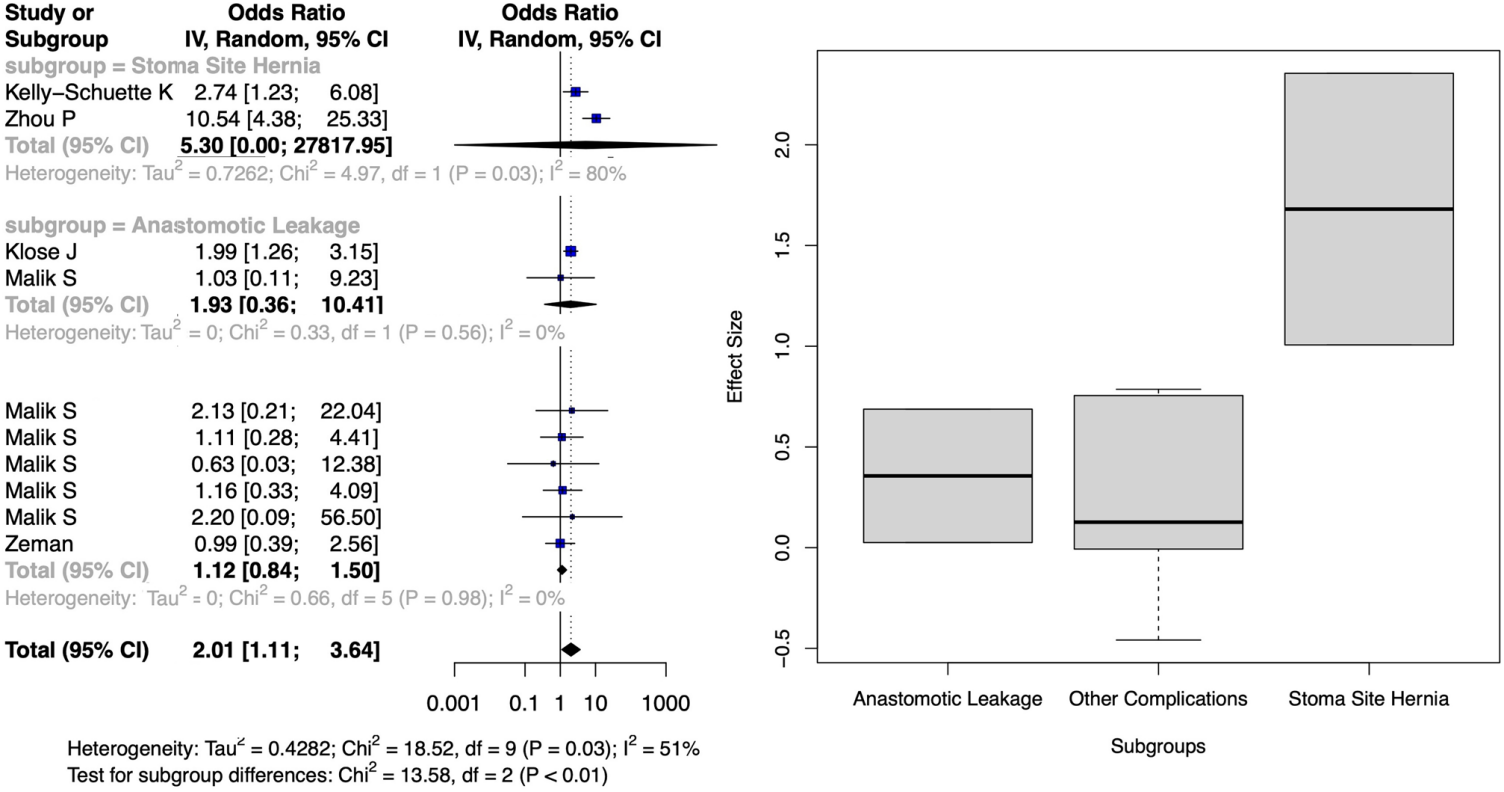
Forest plot of calculated log odds ratios for complications occurring over BMI 30 for included studies. The random-effects meta-analysis model (Inverse Variance method) was used. *Cl* Confidence interval, *df* Degrees of freedom, *IV* Inverse variance, *Std* Standard

A nonsignificant standardized mean difference (SMD) of 0.41 (95% CI − 0.50, 1.31) for BMI-related complications was calculated from five studies [5, 26, 29, 32, 45]. However, subgroup analysis revealed a significant SMD of 0.88 (95% CI 0.34, 1.42) for stoma-site incisional hernia (Fig. 5). Odds ratios (OR) were calculated from five studies each for subsets with BMI > 25 [31, 32, 35, 37, 38] and BMI > 30 [16, 32, 35, 42, 45]. Pooled complications for BMI > 25 did not significantly increase (OR 1.30, 95% CI 0.84, 2.01), but subgroup analysis demonstrated increased risks for stoma-site incisional hernia (OR 4.66, 95% CI 3.54, 6.14) and parastomal hernia (OR 2.41, 95% CI 1.70, 3.40) (Fig. 6). BMI > 30 was associated with a higher overall complication risk (OR 2.01, 95% CI 1.11, 3.64), but subgroup analysis showed no significant differences for anastomotic leakage (OR = 1.93, 95% CI 0.36, 10.41) or stoma-site incisional hernia (OR 5.30, 95% CI 0.00, 27817.95) (Fig. 7).

## Discussion

While initially believed to reduce anastomotic leakage, fecal diversion is now recognized primarily as a strategy to mitigate the consequences of anastomotic leakage. It is predominantly utilized in high-risk anastomoses [40, 46], such as low rectal anastomoses, those in immunocompromised patients, and cases where limited mobilization results in anastomoses under increased tension [4]. Obesity has also been identified as a risk factor for anastomotic leakage [14–16], potentially due to reduced pelvic space and increased technical difficulty [47, 48]. Additionally, obesity is associated with conditions that may necessitate resection and anastomosis, such as diverticulitis and colorectal malignancy [49, 50]. In obese patients, greater intestinal length is required to traverse a thicker abdominal wall, necessitating additional mobilization [51, 52]. Mesenteric fat can obscure visualization of blood vessels [5], and the short, thick, and heavy mesentery complicates the elevation of intestinal limbs for ileostomy formation [48]. Operative times are often prolonged in obese patients [53], serving as a surrogate for increased surgical complexity [54]. Surgical interventions in obese patients are inherently higher risk, with elevated rates of complications, such as surgical site infections and incisional hernia formation [11]. Given these considerations, careful patient selection is essential to balance risks and benefits and avoid additional morbidity. To address technical challenges, some authors recommend stoma creation in the upper abdomen [55] or umbilicus [30] and the use of lubricated wound retractors to enhance conduit mobility [56]. Prolonged postoperative fasting has also been suggested, particularly in cases of super-morbid obesity [51].

### Stoma-site incisional hernia

Incisional hernias develop in up to 48% of previous ileostomy sites following reversal [57], with up to 64% requiring additional surgical intervention [58]. The median time to detection ranges from 6 to 63 months [24, 26, 32, 59, 60], with computed tomography (CT) improving diagnostic yield [61]. All seven (100%) studies reporting this outcome in our review identified BMI as a risk factor for incisional hernia following DLI reversal [24–27, 31, 32, 45]. Our meta-analysis further demonstrated that stoma-site incisional hernias occur more frequently at higher BMI (SMD, 0.88), with a greater than fourfold increase at BMI > 25 (OR, 4.66). No statistically significant difference was observed at BMI > 30, likely due to limited statistical power.

Hernias are more frequently detected in patients treated for malignancy, attributed to longer follow-up durations and increased imaging surveillance [62]. Preventive strategies have been advocated for patients with BMI > 25 [32], which are supported by our findings. Mesh placement at the stoma site has been shown to reduce hernia incidence in several studies [63, 64], including the multicenter ROCSS trial [65], although no difference was observed in the ILEOCLOSE trial [66]. While purse-string closure has been associated with reduced surgical site infections and monofilament sutures may lower incisional hernia risk compared to multifilament sutures [67], skin closure technique, suture material, and surgical site infections do not appear to significantly impact incisional hernia formation [24, 26, 68]. The superiority of open versus laparoscopic approaches remains unclear due to conflicting results in the literature [27, 69]. Short-term weight loss has also been proposed as a preventive measure [25, 39], with newer anti-obesity medications showing potential for prehabilitation.

### Parastomal hernia

Although parastomal hernias were not associated with increased BMI in our review [35], subgroup analysis revealed that BMI > 25 increased the risk (OR, 2.41). Parastomal hernias detected preoperatively or intraoperatively at the time of DLI reversal have been linked to an increased risk of stoma-site incisional hernia formation [24]. A simplified one-stitch technique has been proposed to reduce parastomal hernia risk, offering additional advantages, such as fewer adhesions and shorter operative times during ileostomy creation and reversal [38].

### Peristomal skin complications

Peristomal skin disorders are the most frequent DLI complications, occurring in 42–73% of cases [34, 70]. Two studies (50%) identified increased skin complications at higher BMI. Greater stoma height has been advocated to reduce these complications, although achieving this is more challenging in patients with thicker abdominal walls and increased visceral fat [34]. Ostomy care becomes more difficult, with increased leakage potentially leading to skin irritation.

### Surgical site infections

Following reversal, up to 41% of wounds become infected [71]. A single study (33%) in our review demonstrated an increase in surgical site infections with elevated BMI [41]. While elevated BMI is a known risk factor for surgical site infections, contamination by intestinal contents may play a more significant pathogenic role, minimizing the effect of BMI. Purse-string closure has been shown to reduce surgical site infections compared to conventional linear closure [37], although evidence supporting its routine use in overweight and obese patients remains limited. Other preventive measures include antibiotic implants and prophylactic wound irrigation with povidone-iodine [37], which is currently under investigation in RCTs [72].

### Non-closure resulting in permanent stoma

Temporary stomas are never closed in 3–40% of cases, effectively becoming permanent [33, 73]. A single study (25%) found obesity to increase the likelihood of non-closure [7], potentially due to increased complications precluding further surgery. Several studies report a higher risk of non-closure with open surgery, which may serve as a surrogate for complex surgical histories and medical complications [74]. For nonreversible fecal diversion, permanent colostomy is preferred over permanent ileostomy due to lower risks of dehydration, electrolyte imbalances, and renal dysfunction [75]. Our review suggests that obesity likely does not increase the risk of non-closure, although further studies are needed to confirm this conclusively.

### Stoma retraction

Stoma retraction, though not clearly defined, may complicate 3–17% of ileostomies due to tension on the bowel wall or previous stomal necrosis [76, 77]. It may occur as a result of weight gain, typically after a postoperative period of one month [44]. Obesity has been hypothesized to increase stoma retraction due to a shortened, fatty mesentery and necrosis from inadvertent injury to mesenteric blood vessels [4, 44, 78]. However, our review found no statistical significance in the only study investigating this variable [44].

### Paralytic ileus

DLI may increase the risk of paralytic ileus, although evidence for this relationship is limited [79, 80]. Visceral fat may promote edema, contributing to luminal narrowing and ileus development [39], while increased intraabdominal pressure could result in transient mesenteric ischemia [81, 82]. In our review, one study reported paralytic ileus as an outcome but did not investigate BMI as a variable [30], precluding definitive conclusions.

### Anastomotic leakage

AL occurs in up to 8% of ileostomy reversals [83], with two studies (50%) in our review showing increased anastomotic leakage at higher BMI following the initial anastomosis [16, 43]. This may result from increased technical difficulty or impaired wound healing [47]. Stapled anastomoses may be preferable to hand-sewn anastomoses due to their relative simplicity, shorter operating times, lower morbidity, and anastomotic leakage rates [84, 85].

### High-output ileostomy

High-output ileostomies occur in 16–24% of cases [29], representing the most common reason for readmission following DLI creation [7]. The risk of severe dehydration and electrolyte abnormalities is highest in the early postoperative period [7]. High-output ileostomies did not demonstrate an association with BMI in the two studies evaluating this outcome [7, 29].

### Other complications

Stoma outlet obstruction was increased in one study (100%), potentially due to mechanical twisting, particularly with the laparoscopic approach [40]. While BMI does not appear to significantly impact hospital stay or mortality [35], higher BMI was associated with prolonged operative times [38], delayed closure [36], and increased stoma-specific morbidity scores [4]. These findings likely reflect increased surgical complexity, comorbidities, and other complications.

### Overall complications

Overall complications were increased at higher BMI in two studies (67%) [7, 39], though complications after reversal did not differ in one study (100%) [7]. Our meta-analysis found that overall complications doubled at BMI > 30. Given the heterogeneity among included studies and nonuniform definitions of complications, further large-scale retrospective and prospective studies are warranted to conclusively establish DLI-related morbidity across varying BMI categories. Additionally, risk stratification using categorical BMI definitions is necessary to inform clear clinical guidelines and decision-making.

## Limitations

The heterogeneity of the study designs, diverse underlying pathologies, and various additional surgical procedures, along with other unaccounted confounding factors, may limit the validity of our findings. Multivariate and logistic regression analyses of a larger sample are warranted to provide more robust evidence. Several studies did not report BMI as a variable, and the exclusion of non-English publications further restricted the available data for review and analysis. The mean BMI of the included studies was relatively low, which may have limited the effect size and increased the likelihood of a type II error for several complications.

## Conclusion

Several complications associated with DLI occur more frequently at higher BMI, particularly stoma-site incisional hernia. Other potential BMI-related complications include delayed reversal, stoma outlet obstruction, higher stoma-specific morbidity scores, peristomal skin complications, and longer operative times. The impact of BMI on anastomotic leaks, surgical site infections, and permanent stomas remains inconclusive, while other investigated complications appear unaffected by BMI. If feasible, mesh placement at the ileostomy site should be strongly considered for patients with a BMI > 25 to reduce the risk of stoma-site incisional hernia. Weight loss prior to DLI and reversal should be encouraged.

## Data Availability

All data used for this project are publicly available and accessible online.

## References

[1] Hanna MH, Vinci A, Pigazzi A (2015) Diverting ileostomy in colorectal surgery: when is it necessary? Langenbecks Arch Surg 400(2):145–152. 10.1007/s00423-015-1275-1 25633276 10.1007/s00423-015-1275-1

[2] Horesh N, Hoffman A, Zager Y et al (2019) Value of routine colonic evaluation prior to ileostomy closure. Isr Med Assoc J IMAJ 21(11):728–731 31713360

[3] Rondelli F, Reboldi P, Rulli A et al (2009) Loop ileostomy versus loop colostomy for fecal diversion after colorectal or coloanal anastomosis: a meta-analysis. Int J Colorectal Dis 24(5):479–488. 10.1007/s00384-009-0662-x 19219439 10.1007/s00384-009-0662-x

[4] Zindel J, Gygax C, Studer P et al (2017) A sustaining rod increases necrosis of loop ileostomies: a randomized controlled trial. Int J Colorectal Dis 32(6):875–881. 10.1007/s00384-017-2813-9 28417196 10.1007/s00384-017-2813-9

[5] Yoon YS, Stocchi L, Holubar S et al (2021) When should we add a diverting loop ileostomy to laparoscopic ileocolic resection for primary Crohn’s disease? Surg Endosc 35(6):2543–2557. 10.1007/s00464-020-07670-w 32468260 10.1007/s00464-020-07670-w

[6] Munshi E, Lydrup ML, Buchwald P (2023) Defunctioning stoma in anterior resection for rectal cancer does not impact anastomotic leakage: a national population-based cohort study. BMC Surg 23(1):167. 10.1186/s12893-023-01998-5 37340428 10.1186/s12893-023-01998-5 PMC10283229

[7] Chun LJ, Haigh PI, Tam MS, Abbas MA (2012) Defunctioning loop ileostomy for pelvic anastomoses: predictors of morbidity and nonclosure. Dis Colon Rectum 55(2):167–174. 10.1097/DCR.0b013e31823a9761 22228160 10.1097/DCR.0b013e31823a9761

[8] Chow A, Tilney HS, Paraskeva P, Jeyarajah S, Zacharakis E, Purkayastha S (2009) The morbidity surrounding reversal of defunctioning ileostomies: a systematic review of 48 studies including 6,107 cases. Int J Colorectal Dis 24(6):711–723. 10.1007/s00384-009-0660-z 19221766 10.1007/s00384-009-0660-z

[9] Benoist S, Panis Y, Alves A, Valleur P (2000) Impact of obesity on surgical outcomes after colorectal resection. Am J Surg 179(4):275–281. 10.1016/s0002-9610(00)00337-8 10875985 10.1016/s0002-9610(00)00337-8

[10] Balentine CJ, Wilks J, Robinson C et al (2010) Obesity increases wound complications in rectal cancer surgery. J Surg Res 163(1):35–39. 10.1016/j.jss.2010.03.012 20605591 10.1016/j.jss.2010.03.012

[11] Gurunathan U, Ramsay S, Mitrić G, Way M, Wockner L, Myles P (2017) Association between obesity and wound infection following colorectal surgery: systematic review and meta-analysis. J Gastrointest Surg 21(10):1700–1712. 10.1007/s11605-017-3494-y 28785932 10.1007/s11605-017-3494-y

[12] Young MT, Hwang GS, Menon G et al (2015) Laparoscopic versus open loop ileostomy reversal: is there an advantage to a minimally invasive approach? World J Surg 39(11):2805–2811. 10.1007/s00268-015-3186-2 26272594 10.1007/s00268-015-3186-2

[13] De Robles MS, Bakhtiar A, Young CJ (2019) Obesity is a significant risk factor for ileostomy site incisional hernia following reversal. ANZ J Surg 89(4):399–402. 10.1111/ans.14983 30684304 10.1111/ans.14983

[14] Frasson M, Flor-Lorente B, Rodríguez JLR et al (2015) Risk factors for anastomotic leak after colon resection for cancer: multivariate analysis and nomogram from a multicentric, prospective, national study with 3193 patients. Ann Surg 262(2):321–330. 10.1097/SLA.0000000000000973 25361221 10.1097/SLA.0000000000000973

[15] Matthiessen P, Henriksson M, Hallböök O, Grunditz E, Norén B, Arbman G (2008) Increase of serum C-reactive protein is an early indicator of subsequent symptomatic anastomotic leakage after anterior resection. Colorectal Dis 10(1):75–80. 10.1111/j.1463-1318.2007.01300.x 17666099 10.1111/j.1463-1318.2007.01300.x

[16] Klose J, Tarantino I, Von Fournier A et al (2018) A nomogram to predict anastomotic leakage in open rectal surgery—hope or hype? J Gastrointest Surg 22(9):1619–1630. 10.1007/s11605-018-3782-1 29777457 10.1007/s11605-018-3782-1

[17] Page MJ, McKenzie JE, Bossuyt PM et al (2021) The PRISMA 2020 statement: an updated guideline for reporting systematic reviews. BMJ. 10.1136/bmj.n71 33782057 10.1136/bmj.n71 PMC8005924

[18] Sadiq KO, Ruiz Cota P, Lakshminarayanan S, Marquez Castillo E (2024). Postoperative morbidity of diverting loop ileostomies in patients with elevated BMI. National Institute for Health and Care Research. https://www.crd.york.ac.uk/PROSPERO/view/CRD42024581114

[19] Wells GA, Shea B, O’Connell D et al The Newcastle-Ottawa Scale (NOS) for assessing the quality of nonrandomised studies in meta-analyses. 2000. https://scholar.archive.org/work/zuw33wskgzf4bceqgi7opslsre/access/wayback/http://www3.med.unipmn.it/dispense_ebm/2009-2010/Corso%20Perfezionamento%20EBM_Faggiano/NOS_oxford.pdf. Accessed 11 March 2025

[20] Agency for Healthcare Research and Quality. Newcastle-Ottawa Quality Assessment Form for Cohort Studies. https://www.ncbi.nlm.nih.gov/books/NBK115843/bin/appe-fm3.pdf.

[21] Sterne JAC, Savović J, Page MJ et al (2019) RoB 2: a revised tool for assessing risk of bias in randomised trials. BMJ. 10.1136/bmj.l4898 31462531 10.1136/bmj.l4898

[22] Cai S, Zhou J, Pan J (2021) Estimating the sample mean and standard deviation from order statistics and sample size in meta-analysis. Stat Methods Med Res 30(12):2701–2719. 10.1177/09622802211047348 34668458 10.1177/09622802211047348

[23] Beheshti A, Chavanon ML, Christiansen H (2020) Emotion dysregulation in adults with attention deficit hyperactivity disorder: a meta-analysis. BMC Psychiatry 20(1):120. 10.1186/s12888-020-2442-7 32164655 10.1186/s12888-020-2442-7 PMC7069054

[24] Barranquero AG, Tobaruela E, Bajawi M, Muñoz P, Die Trill J, Garcia-Perez JC (2020) Incidence and risk factors for incisional hernia after temporary loop ileostomy closure: choosing candidates for prophylactic mesh placement. Hernia 24(1):93–98. 10.1007/s10029-019-02042-3 31494806 10.1007/s10029-019-02042-3

[25] Bloomfield I, Dobson B, Von Papen M, Clark D (2022) Incisional hernia following ileostomy closure: who’s at risk? The Gold Coast experience. ANZ J Surg 92(1–2):146–150. 10.1111/ans.17359 34791754 10.1111/ans.17359

[26] Brook AJ, Mansfield SD, Daniels IR, Smart NJ (2018) Incisional hernia following closure of loop ileostomy: the main predictor is the patient, not the surgeon. The Surg 16(1):20–26. 10.1016/j.surge.2016.03.004 10.1016/j.surge.2016.03.004 27161097

[27] Eklöv K, Viktorsson FZ, Frosztega E, Bringman S, Nygren J, Everhov ÅH (2020) Hernia at the stoma site after loop ileostomy reversal. Int J Colorectal Dis 35(5):887–895. 10.1007/s00384-020-03542-w 32124049 10.1007/s00384-020-03542-w

[28] Eray İC, Rencüzoğullari A, Yalav O, Topal U, Saritaş AG, Dalci K (2019) Incidence of permanent stoma after rectal cancer surgery and its risk factors. Cukurova Med J 44(4):1463–1467. 10.17826/cumj.529941

[29] Hiramoto Y, Kawahara H, Matsumoto T, Takeda M, Misawa T, Yanaga K (2019) Preoperative neutrophil–lymphocyte ratio is a predictor of high-output ileostomy after colorectal surgery. Anticancer Res 39(6):3265–3268. 10.21873/anticanres.13468 31177177 10.21873/anticanres.13468

[30] Ishiguro S, Komatsu S, Ando K et al (2017) Feasibility of umbilical loop ileostomy in overweight and obese patients with rectal cancer during laparoscopic surgery: preliminary results. Asian J Endosc Surg 10(1):79–82. 10.1111/ases.12316 28045234 10.1111/ases.12316

[31] Kaneko T, Funahashi K, Ushigome M et al (2019) Incidence of and risk factors for incisional hernia after closure of temporary ileostomy for colorectal malignancy. Hernia 23(4):743–748. 10.1007/s10029-018-1855-4 30426253 10.1007/s10029-018-1855-4

[32] Kelly-Schuette K, Wilkes A, Kyriakakis R, Ogilvie J (2020) Predictors of hernia after loop ileostomy closure: a single-center retrospective review. Int J Colorectal Dis 35(9):1695–1702. 10.1007/s00384-020-03637-4 32451647 10.1007/s00384-020-03637-4

[33] Kim MJ, Kim YS, Park SC et al (2016) Risk factors for permanent stoma after rectal cancer surgery with temporary ileostomy. Surgery 159(3):721–727. 10.1016/j.surg.2015.09.011 26490725 10.1016/j.surg.2015.09.011

[34] Maeda S, Ouchi A, Komori K et al (2021) Risk factors for peristomal skin disorders associated with temporary ileostomy construction. Surg Today 51(7):1152–1157. 10.1007/s00595-020-02209-x 33569690 10.1007/s00595-020-02209-x

[35] Malik S, Memon AI, Kumar H, Masroor A, Zeb M, Gul S (2023) Original article early closure of loop ileostomies in typhoid perforation. Med Forum 34(8)

[36] Manterola C, Claros N (2023) Early vs. standard loop ileostomy closure an unmatched case-control study. Int J Morphol 41(6):1863–1869. 10.4067/S0717-95022023000601863

[37] Nyandoro MG, Seow YT, Stein J, Theophilus M (2023) Single-centre experience of loop ileostomy closure: a retrospective comparison of conventional-linear closure and purse-string closure on surgical-site-infection rates. ANZ J Surg 93(3):629–635. 10.1111/ans.18083 36197316 10.1111/ans.18083

[38] Pei W, Cui H, Liu Z et al (2021) One-stitch method vs traditional method of protective loop ileostomy for rectal cancer the impact of BMI obesity. J Cancer Res Clin Oncol 147(9):2709–2719. 10.1007/s00432-021-03556-z 33606093 10.1007/s00432-021-03556-z PMC11801953

[39] Saito Y, Akura YT, Hinoi T, Egi H, Tashiro H, Ohdan H (2014) Body mass index as a predictor of postoperative complications in loop ileostomy closure after rectal resection in Japanese patients. Hiroshima J Med Sci 63(4):33–8 25707091

[40] Tamura K, Matsuda K, Yokoyama S et al (2019) Defunctioning loop ileostomy for rectal anastomoses: predictors of stoma outlet obstruction. Int J Colorectal Dis 34(6):1141–1145. 10.1007/s00384-019-03308-z 31055627 10.1007/s00384-019-03308-z

[41] Von Savigny C, Juratli MA, Koch C, Gruber-Rouh T, Bechstein WO, Schreckenbach T (2023) Short-term outcome of diverting loop ileostomy reversals performed by residents: a retrospective cohort prognostic factor study. Int J Colorectal Dis 38(1):108. 10.1007/s00384-023-04390-0 37084093 10.1007/s00384-023-04390-0 PMC10121496

[42] Zeman M, Czarnecki M, Chmielarz A, Idasiak A, Grajek M, Czarniecka A (2020) Assessment of the risk of permanent stoma after low anterior resection in rectal cancer patients. World J Surg Oncol 18(1):207. 10.1186/s12957-020-01979-5 32795302 10.1186/s12957-020-01979-5 PMC7427951

[43] Zhang B, Zhuo GZ, Zhao K et al (2022) Cumulative incidence and risk factors of permanent stoma after intersphincteric resection for ultralow rectal cancer. Dis Colon Rectum 65(1):66–75. 10.1097/DCR.0000000000002036 34882629 10.1097/DCR.0000000000002036

[44] Uchino M, Ikeuchi H, Bando T, Chohno T, Sasaki H, Horio Y (2017) Is an ostomy rod useful for bridging the retraction during the creation of a loop ileostomy? A randomized control trial. World J Surg 41(8):2128–2135. 10.1007/s00268-017-3978-7 28299472 10.1007/s00268-017-3978-7

[45] Zhou P, Hrabe J, Byrn J (2016) A retrospective, single-institution review of loop ileostomy reversal outcomes. Ostomy Wound Manage 62(8):22–33 27564436

[46] Chang YWW, Davenport D, Dugan A, Patel JA (2020) Significant morbidity is associated with proximal fecal diversion among high-risk patients who undergo colectomy: a NSQIP analysis. Am J Surg 220(4):830–835. 10.1016/j.amjsurg.2020.05.007 32482294 10.1016/j.amjsurg.2020.05.007

[47] Doyle SL, Lysaght J, Reynolds JV (2010) Obesity and post-operative complications in patients undergoing non-bariatric surgery. Obes Rev Off J Int Assoc Study Obes 11(12):875–886. 10.1111/j.1467-789X.2009.00700.x 10.1111/j.1467-789X.2009.00700.x 20025695

[48] Esen E, Aytac E, Aydinli HH et al (2022) Ileal pouch excision can be performed with similar outcomes in obese patients compared to nonobese counterparts: an assessment from American College of Surgeons National Surgical Quality Improvement Program. Am Surg 88(12):2857–2862. 10.1177/00031348211011121 33856901 10.1177/00031348211011121

[49] Ma W, Jovani M, Liu PH et al (2018) Association between obesity and weight change and risk of diverticulitis in women. Gastroenterology 155(1):58-66.e4. 10.1053/j.gastro.2018.03.057 29614301 10.1053/j.gastro.2018.03.057 PMC6035062

[50] Bardou M, Barkun AN, Martel M (2013) Obesity and colorectal cancer. Gut 62(6):933–947. 10.1136/gutjnl-2013-304701 23481261 10.1136/gutjnl-2013-304701

[51] Fujimoto N, Ogino T, Miyoshi N, Uemura M, Doki Y, Eguchi H (2024) Avoiding stoma creation due to super-morbid obesity: a report of two surgical cases of colorectal cancer. Int J Surg Case Rep 114:109171. 10.1016/j.ijscr.2023.109171 38113563 10.1016/j.ijscr.2023.109171 PMC10772238

[52] Beck S (2011) Stoma issues in the obese patient. Clin Colon Rectal Surg 24(04):259–262. 10.1055/s-0031-1295689 23204941 10.1055/s-0031-1295689 PMC3311493

[53] Panteleimonitis S, Popeskou S, Harper M et al (2018) Minimally invasive colorectal surgery in the morbid obese: does size really matter? Surg Endosc 32(8):3486–3494. 10.1007/s00464-018-6068-5 29362912 10.1007/s00464-018-6068-5 PMC6061053

[54] Cheng H, Chen BPH, Soleas IM, Ferko NC, Cameron CG, Hinoul P (2017) Prolonged operative duration increases risk of surgical site infections: a systematic review. Surg Infect 18(6):722–735. 10.1089/sur.2017.089 10.1089/sur.2017.089 PMC5685201 28832271

[55] Cataldo PA (2008) Technical tips for stoma creation in the challenging patient. Clin Colon Rectal Surg 21(1):17–22. 10.1055/s-2008-1055317 20011392 10.1055/s-2008-1055317 PMC2780186

[56] Meagher AP, Owen G, Gett R (2009) Multimedia article. An improved technique for end stoma creation in obese patients. Dis Colon Rectum 52(3):531–533. 10.1007/DCR.0b013e31819a2441 19333058 10.1007/DCR.0b013e31819a2441

[57] Bhangu A, Nepogodiev D, Futaba K (2012) West midlands research collaborative systematic review and meta-analysis of the incidence of incisional hernia at the site of stoma closure. World J Surg 36(5):973–983 22362042 10.1007/s00268-012-1474-7

[58] Sharp SP, Francis JK, Valerian BT, Canete JJ, Chismark AD, Lee EC (2015) Incidence of ostomy site incisional hernias after stoma closure. Am Surg 81(12):1244–1248 26736162

[59] El-Hussuna A, Lauritsen M, Bülow S (2012) Relatively high incidence of complications after loop ileostomy reversal. Dan Med J 59(10):A4517 23158893

[60] Saha AK, Tapping CR, Foley GT et al (2009) Morbidity and mortality after closure of loop ileostomy. Colorectal Dis Off J Assoc Coloproctology G B Irel 11(8):866–871. 10.1111/j.1463-1318.2008.01708.x 10.1111/j.1463-1318.2008.01708.x 19175627

[61] Cingi A, Cakir T, Sever A, Aktan AO (2006) Enterostomy site hernias: a clinical and computerized tomographic evaluation. Dis Colon Rectum 49(10):1559–1563. 10.1007/s10350-006-0681-4 17120189 10.1007/s10350-006-0681-4

[62] Baucom RB, Beck WC, Holzman MD, Sharp KW, Nealon WH, Poulose BK (2014) The importance of surgeon-reviewed computed tomography for incisional hernia detection: a prospective study. Am Surg 80(7):720–722 24987907

[63] van den Hil LCL, van Steensel S, Schreinemacher MHF, Bouvy ND (2019) Prophylactic mesh placement to avoid incisional hernias after stoma reversal: a systematic review and meta-analysis. Hernia J Hernias Abdom Wall Surg 23(4):733–741. 10.1007/s10029-019-01996-8 10.1007/s10029-019-01996-8 PMC6661031 31302788

[64] Mohamedahmed AYY, Stonelake S, Zaman S, Hajibandeh S (2020) Closure of stoma site with or without prophylactic mesh reinforcement: a systematic review and meta-analysis. Int J Colorectal Dis 35(8):1477–1488. 10.1007/s00384-020-03681-0 32588121 10.1007/s00384-020-03681-0

[65] Bhangu A, Nepogodiev D, Ives N et al (2020) Prophylactic biological mesh reinforcement versus standard closure of stoma site (ROCSS): a multicentre, randomised controlled trial. The Lancet 395(10222):417–426. 10.1016/S0140-6736(19)32637-6 10.1016/S0140-6736(19)32637-6 PMC7016509 32035551

[66] Villanueva Figueredo B Ileoclose trial. Estudio unicéntrico aleatorizado de profilaxis de la eventración en el cierre de ileostomías. Published online 2020.

[67] Patel SV, Paskar DD, Nelson RL, Vedula SS, Steele SR (2017) Closure methods for laparotomy incisions for preventing incisional hernias and other wound complications. Cochrane Database Syst Rev 11(11):CD005661. 10.1002/14651858.CD005661.pub2 29099149 10.1002/14651858.CD005661.pub2 PMC6486019

[68] Henriksen NA, Deerenberg EB, Venclauskas L, Fortelny RH, Miserez M, Muysoms FE (2018) Meta-analysis on materials and techniques for laparotomy closure: the MATCH review. World J Surg 42(6):1666–1678. 10.1007/s00268-017-4393-9 29322212 10.1007/s00268-017-4393-9

[69] Calvo Espino P, Sánchez Movilla A, Alonso Sebastian I et al (2022) Incidence and risk factors of delayed development for stoma site incisional hernia after ileostomy closure in patients undergoing colorectal surgery with temporary ileostomy. Acta Chir Belg 122(1):41–47. 10.1080/00015458.2020.1846941 33176613 10.1080/00015458.2020.1846941

[70] Carlsson E, Fingren J, Hallén AM, Petersén C, Lindholm E (2016) The prevalence of ostomy-related complications 1 year after ostomy surgery: a prospective, descriptive, clinical study. Ostomy Wound Manage 62(10):34–48 27768579

[71] Lee JR, Kim YW, Sung JJ et al (2011) Conventional linear versus purse-string skin closure after loop ileostomy reversal: comparison of wound infection rates and operative outcomes. J Korean Soc Coloproctol 27(2):58. 10.3393/jksc.2011.27.2.58 21602963 10.3393/jksc.2011.27.2.58 PMC3092076

[72] Maemoto R, Noda H, Ichida K et al (2021) Superiority trial comparing intraoperative wound irrigation with aqueous 10% povidone-iodine to saline for the purpose of reducing surgical site infection after elective gastrointestinal surgery: study protocol for a randomised controlled trial. BMJ Open 11(6):e051374. 10.1136/bmjopen-2021-051374 34135056 10.1136/bmjopen-2021-051374 PMC8211046

[73] Sier MF, Van Gelder L, Ubbink DT, Bemelman WA, Oostenbroek RJ (2015) Factors affecting timing of closure and non-reversal of temporary ileostomies. Int J Colorectal Dis 30(9):1185–1192. 10.1007/s00384-015-2253-3 26054385 10.1007/s00384-015-2253-3 PMC4553149

[74] Li C, Qin X, Yang Z et al (2021) A nomogram to predict the incidence of permanent stoma in elderly patients with rectal cancer. Ann Transl Med 9(4):342–342. 10.21037/atm-21-29 33708969 10.21037/atm-21-29 PMC7944294

[75] He F, Tang C, Yang F et al (2024) Preoperative risk factors and cumulative incidence of temporary ileostomy non-closure after sphincter-preserving surgery for rectal cancer: a meta-analysis. World J Surg Oncol 22(1):94. 10.1186/s12957-024-03363-z 38610000 10.1186/s12957-024-03363-z PMC11010286

[76] Cottam J, Richards K, Hasted A, Blackman A (2007) Results of a nationwide prospective audit of stoma complications within 3 weeks of surgery. Colorectal Dis 9(9):834–838. 10.1111/j.1463-1318.2007.01213.x 17672873 10.1111/j.1463-1318.2007.01213.x

[77] Shellito PC (1998) Complications of abdominal stoma surgery. Dis Colon Rectum 41(12):1562–1572. 10.1007/BF02237308 9860339 10.1007/BF02237308

[78] Leenen LP, Kuypers JH (1989) Some factors influencing the outcome of stoma surgery. Dis Colon Rectum 32(6):500–504. 10.1007/BF02554506 2791788 10.1007/BF02554506

[79] Chapuis PH, Bokey L, Keshava A et al (2013) Risk factors for prolonged ileus after resection of colorectal cancer: an observational study of 2400 consecutive patients. Ann Surg 257(5):909–915. 10.1097/SLA.0b013e318268a693 23579542 10.1097/SLA.0b013e318268a693

[80] Millan M, Biondo S, Fraccalvieri D, Frago R, Golda T, Kreisler E (2012) Risk factors for prolonged postoperative ileus after colorectal cancer surgery. World J Surg 36(1):179–185. 10.1007/s00268-011-1339-5 22083434 10.1007/s00268-011-1339-5

[81] Jafari MD, Halabi WJ, Jafari F et al (2013) Morbidity of diverting ileostomy for rectal cancer: analysis of the american college of surgeons national surgical quality improvement program. Am Surg. 10.1177/000313481307901016 24160794

[82] Khoo RE, Cohen MM, Chapman GM, Jenken DA, Langevin JM (1994) Loop ileostomy for temporary fecal diversion. Am J Surg 167(5):519–522. 10.1016/0002-9610(94)90249-6 8185041 10.1016/0002-9610(94)90249-6

[83] Faunø L, Rasmussen C, Sloth KK, Sloth AM, Tøttrup A (2012) Low complication rate after stoma closure consultants attended 90% of the operations. Colorectal Dis 14(8):499–505. 10.1111/j.1463-1318.2012.02991.x 10.1111/j.1463-1318.2012.02991.x 22340709

[84] Hasegawa H, Radley S, Morton DG, Keighley MR (2000) Stapled versus sutured closure of loop ileostomy: a randomized controlled trial. Ann Surg 231(2):202–204. 10.1097/00000658-200002000-00008 10674611 10.1097/00000658-200002000-00008 PMC1420987

[85] Shelygin YA, Chernyshov SV, Rybakov EG (2010) Stapled ileostomy closure results in reduction of postoperative morbidity. Tech Coloproctology 14(1):19–23. 10.1007/s10151-009-0550-y 10.1007/s10151-009-0550-y 20013018

